# Behavioral and pharmacological effects of phytochemicals in *Cinnamomum verum* bark extract for prevention of experimental murine coccidiosis

**DOI:** 10.3389/fvets.2025.1715869

**Published:** 2025-11-20

**Authors:** Mutee Murshed, Hind Alzaylaee, Khalid Elfaki Ibrahim, Aiman Ammari, Hani Ahmed, Saleh Al-Quraishy

**Affiliations:** 1 Department of Zoology, College of Science, King Saud University, Riyadh, Saudi Arabia; 2 Department of Biology, College of Science, Princess Nourah bint Abdulrahman University, Riyadh, Saudi Arabia; 3 School of Pharmaceutical Science, Nanchang University, Nanchang, China

**Keywords:** extraction, pharmaceutical industry, antioxidant, biological activity, plants, Eimeriosis, *Cinnamomum verum* bark, FTIR

## Abstract

**Objectives:**

Natural products hold significant potential in combating parasitic infections. Parasites belonging to the genus *Eimeria* are responsible for coccidiosis, which affects a wide variety of livestock worldwide. The emergence of drug resistance to coccidia has prompted renewed efforts to discover novel chemicals for alternative therapeutic techniques. This research was conducted to evaluate the effectiveness of *Cinnamomum verum* bark extract (CVBE) on behavior and the treatment of eimeriosis in mice.

**Methods:**

The study involved the examination of five groups of mice, with *E. papillate* sporulation oocysts (1 × 10^3^) being utilized to infect three groups by experimental means. The first group was the control, the second group was administered at a dosage of 100 mg/kg of CVBE only to test toxicity, and the third was the infected positive control. The fourth group was treated daily with 100 mg/kg of CVBE, while the fifth group received 50 mg/kg of amprolium via gavage. All mice were sedated using CO₂ and subsequently dissected for sample taking. Results FT-IR analysis, conducted using a Thermo Scientific optical spectrometer, revealed that CVBE contains 18 different phytochemical components. The administration of CVBE led to a significant decrease in the number of oocysts produced in the feces. Additionally, the parasite stages exhibited morphometric changes, with decreases in the measures compared with those of the infected but not treated mice. This improvement was accompanied by large increases in the number of goblet cells, which provided additional support for the effectiveness of CVBE as anticoccidial agents.

**Conclusion:**

The findings indicate that *Cinnamomum verum* bark extract has effective effects in reducing the shedding of cysts, possesses anticoccidial, antioxidant, and anti-inflammatory activity against jejunum injury induced by the parasite *Eimeria papillata*.

## 1 Introduction

Coccidiosis is an intestinal disease that affects a wide variety of animals and affects the health and function of several vertebrates; it is caused by *Eimeria* spp. in birds and cattle or Isospora in felids and canids (1) The annual propagation rate of coccidiosis in domestic avians in Riyadh, Saudi Arabia, is 80% (2). These protozoan parasites present a threat to both animals and humans. They cause substantial harm to the economy of the cattle sector (3). *Eimeria* swiftly multiply within host cells, causing harm to their digestive systems (4). Disease symptoms include diarrhea, which varies from watery to hemorrhagic mucoid and is accompanied by feed malabsorption and reduced feed conversion efficiency, resulting in weight loss and, in severe instances, mortality (5). Mice infected with *Eimeria papillata* exhibit infections in their jejunum. This infection significantly affects the intestinal mucosa (6). Mice are the most commonly employed mammalian model in biomedical research. Thus, *E. papillata* serves as an exemplary model for the study of avian coccidiosis (7). Because parasites are becoming more resistant to antiparasitic drugs, treatment doses and residues in animal products have increased. This has made consumers want products without residues and led many researchers to suggest and develop other agents (8).

The growing interest in safe and effective options for coccidiosis management has resulted in the utilization of plant extracts, essential oils, and traditional medicinal preparations (9). Medicinal plants serve as the fundamental source for all alternative or natural medical systems and are considered therapeutic methods for acquiring potent chemical constituents (10). These medicines demonstrate organ-protective properties in hosts infected with Eimeria and specifically target parasites (11).

In recent decades, medicinal plant extracts have been shown to effectively safeguard animals from coccidia-induced harm, albeit without enhancing performance (12) The organic acid combinations suppressed both *in vitro* and *in vivo* pathogens, diminished inflammatory oxidative stress, and boosted the immune response by lowering reactive oxygen species, increasing manganese superoxide dismutase, and elevating short-chain fatty acid levels (13).

*Cinnamomum verum*, a natural product of the Lauraceae family, is abundant in several bioactive chemicals with diverse medicinal qualities. It comprises numerous secondary plant chemicals, including cinnamaldehyde, carvacrol, eugenol, camphor, and proanthocyanidins, as well as essential macro- and micronutrients (14). Owing to these bioactive components, botanicals, including essential oils, have demonstrated promising anticoccidial efficacy (15). These bioactive molecules exhibit antioxidant capabilities, hence mitigating the oxidative damage caused by *Eimeria* (16). *C. verum*, usually referred to as “cinnamon,” is a widely utilized spice recognized for its diverse therapeutic and pharmacological attributes, including antioxidant, anti-inflammatory, and antibacterial capabilities (17). Considering these characteristics, the present investigation was undertaken to evaluate the *in vivo* anticoccidial efficacy of *C. verum* essential against *E. papillata* infection and its impact on metabolic markers in mice.

## 2 Manuscript formatting

### 2.1 Extraction of *Cinnamomum verum* bark

The collection of *C. verum* bark came from spice markets in Riyadh. The Botany Department confirmed the taxonomic identity of the materials with number 24649. Once the bark was dried at 40 °C, it was chopped into small pieces. An electric blender was subsequently used to powder the bark. This powder was extracted by macerating it with 70% ethanol (18). We continuously mixed this mixture and then placed it in an incubator at 4 °C for 24 h. After centrifugation for 15 min at 5000 rpm, we carried out filtration. We concentrated the supernatant at 50 °C under reduced pressure via a rotary evaporator. To prepare dosages of CVBE, 100, 50, and 25 mg/kg, the powdered *C. verum* extract that was obtained was freeze-dried and stored at −80 °C.

### 2.2 Infrared spectroscopy of the CVBE

Upon extraction, a small fraction of the material was homogenized by blending it with an abundant amount of potassium bromide powder (1:99 wt%). The material subsequently underwent a coarse crushing procedure before being introduced into a pellet-forming die. The infrared spectrum was examined via a Thermo Scientific optical spectrometer [NICOLET 6700 Fourier transform infrared spectroscopy (FT-IR)]. This showed the forecasting of the most likely constituent classes. The maximum quantity of absorbed waves is represented by the term “wavenumber” (cm^−1^). Spectra were acquired at 25 °C, with a resolution of 4 cm^−1^, covering a spectral range from 4,000 cm^−1^ to 400 cm^−1^.

### 2.3 Design of the experiment

*Eimeria papillata* was obtained mainly from unpopulated oocysts by Prof. Mehlhorn at the University of Duesseldorf (Duesseldorf, Germany). Forty-five female BALB/C mice were obtained from an animal facility in Riyadh, Saudi Arabia. The mice were 9 ± 1-week-old, weighed 20 ± 2 g, and were maintained in settings that were free of any pathogens. Food and water were provided ad libitum, and the mice were kept on a 12-h light/dark cycle at a regulated temperature (26 ± 2 °C) and 65 ± 5% humidity. In addition, the feces of the mice were examined to guarantee that the animals were free of intestinal diseases, especially coccidia. A preliminary dose–response study was performed to determine the optimal dose of CVBE used 20 mice (injured 1 × 10^3^ sporulated oocysts) distributed over four concentrations (12.5, 25, 50, and 100 mg/kg). In the final experiment, the animals were separated into five groups (5 mice/group), and an oral dose of 1 × 10^3^ sporulated *E. papillata* oocysts was administered to all groups, except the first (negative control) and second groups (noninfected and treated with CVBE at a dose of 100 mg/kg). The positive control mice, which were infected but not treated, composed the third group. A dose of 100 mg/kg CVBE was administered to infected mice in the fourth group. In conclusion, the fifth group consisted of mice that had been infected and treated with amprolium at a dosage of 50 mg/kg. All mice were sedated using CO₂ and subsequently dissected for sample taking. The mice are placed in a sealed glass cage connected by a tube to a CO₂ cylinder, and a dose enough to anesthetize the mouse is given, proportionate to the mouse’s weight.

### 2.4 Evaluation of Anticoccidial activity

The anticoccidial index (ACI) was used to evaluate the preventive efficiency of CNBE against coccidia. Anticoccidial characteristics, including bloody diarrhea, oocyst value (OV), relative weight gain (RWG), lesion value (LV), and survival rate (SR), were assessed to compute the anticoccidial index (ACI) via the method of Habibi et al. (19).

ACIvalue=(RWG+SR)−(LV+OV)

ACI values less than 120 were deemed to indicate inactive anticoccidial action, although ACI values typically range from 0–200 (20) Consequently, ACI values ranging from 120–140, 140–160, 160–180, or over 180 were categorized as mild, moderate, marked, or outstanding. Bloody diarrhea in the mice was documented in the morning with daily veterinary surveillance and quantification of blood fragments in the feces during a period of 3 to 5 days post-infection. The feces were eliminated daily following each inspection to reveal the fresh bloody feces. The digits 0, 1, 2, 3, and 4 correspond to the respective values of the bloody fragments in the feces of each group (21).

Clinical symptoms and death were evaluated and documented daily following infection. Equation 5 was subsequently employed to determine the survival rate.

$$\begin{array}{l} \text{Survival rate}\;(\mathrm{SR}\%)=\\ {\left(\text{number of surviving mice}/\text{initial number of mice}\right)}^{*}\;100 \end{array}$$

$$\begin{array}{l} \text{Oocyst reduced}\%=\mathrm{No}.\mathrm{OPG}\;\text{of infected}-\\ \mathrm{No}.\mathrm{OPG}\;\text{of experimental group}/\mathrm{No}.\mathrm{OPG}\;{\text{of infected}}^{*}\;100 \end{array}$$

On the fifth day, the experiment ended at 5 DPI, during which all the mice (with a sample size of 1 mouse per duplicate, *n* = 5 mice per group) were subjected to 3 h of food deprivation, and their drinking water was provided ad libitum. The mice were subsequently euthanized and autopsied in compliance with the Animal Welfare Act. Following slaughter by severing the jugular vein, the mice were thoroughly bled, subsequently deskinned and eviscerated. The jejunum of each mouse was excised and analyzed. Lesion values were assessed based on the macroscopic lesions found during necropsy, utilizing a scale of 0–4, which was determined by the severity of the lesion and atrophy of the jejunum (including fluid buildup, epithelial coloration, and overall intestinal appearance). Zero indicates the absence of lesions, denoting a ‘normal condition’, whereas one signifies mild lesions characterized by small, scattered petechiae and normal jejunal wall thickness. Two findings indicate significant lesions: many petechiae and reduced jejunal wall thickness. Three indicates severe lesions characterized by significant hemorrhaging, distended jejunal walls, and morphological alterations of the jejunum; four denotes more severe lesions, including discoloration, blood clots, and atrophy of the jejunum, as well as pronounced hypertrophy of the jejunal walls, culminating in fatalities due to coccidiosis. Additionally, the jejunum lengths were documented as the mean ± SEM (in cm) to evaluate the degree of atrophy.

### 2.5 Oocyst number

On the fifth day post infection (p.i.), we isolated each mouse from the third, fourth, and fifth groups into individual cages (*n* = 5 mice) to obtain fresh fecal pellets. Recent fecal samples were obtained from the mice. The feces were suspended in a saturated saline solution containing 0.9% sodium chloride, with each sample meticulously blended and disseminated in 10 mL of water. The weight-to-volume ratio was 1:10. A calculation was conducted to determine the average quantity of oocysts excreted by both the treated and untreated groups via a glass microscope slide and sanitized square microscope coverslips measuring 18 × 18 mm via an Olympus optical microscope (Olympus Corporation, Tokyo, Japan) with a digital camera (22). The results were quantified as oocysts/g (OPG). The rates of reduction in oocyst generation and oocyst values were determined as specified by Smith et al. (23).

$$\begin{array}{l} \text{Oocyst value}\;(\%)=\mathrm{No}.\mathrm{OPG}\;\text{output of every}\\ \text{group}/\mathrm{No}.\mathrm{OPG}\;\text{output of infected}\times 100 \end{array}$$

$$\begin{array}{l} \text{Oocyst reduced}\%=\mathrm{No}.\mathrm{OPG}\;\text{of infected}-\\ \mathrm{No}.\mathrm{OPG}\;\text{of experimental group}/\mathrm{No}.\mathrm{OPG}\;{\text{of infected}}^{*}100 \end{array}$$

### 2.6 Histopathological examination and count of parasitic stages

The jejunum was subsequently separated with a phosphate-buffered saline solution (pH = 7.5) and promptly fixed in a 10% neutral buffered formalin solution at 24 °C for 1 day. The tissue samples were dehydrated via ethanol in increasing order, cleared with xylene, fixed in paraffin wax, and sectioned into 5 μm slices on glass slides. The samples were deparaffinized with xylene, and the tissue sections were stained with hematoxylin and eosin (H&E) and Masson’s trichrome (MTC). The slides were fixed and allowed to dry in anticipation of the examination. An Olympus compound microscope and a digital camera (Olympus 6.0, Tokyo, Japan) were used to capture images and analyze the samples (24).

### 2.7 The enumeration of goblet cells

Alcian blue was used to stain 5 μm thick tissue sections for 5 min, followed by deparaffinization and processing, to quantify the number of goblet cells. The samples were repeatedly cleansed with distilled water, subsequently dehydrated with decreasing concentrations of water, and then deparaffinized with xylene and ethanol to remove moisture. The jejunum of each animal was examined for the quantity of goblet cells in a minimum of ten properly orientated villous–crypt units (VCUs). The findings are presented as the average count of goblet cells observed in each villus (25).

### 2.8 Oxidative stress in jejunal

The jejunum was prepared after weighing from each of the noninfected, nontreated, and infected groups of mice to assess alterations in the oxidation state. The sample was promptly homogenized in 50 mM phosphate-buffered saline (PBS) with a pH exceeding 7.4, followed by centrifugation at 5000 × g for 15 min at 4 °C. A 10% (wt/v) jejunum homogenate was obtained after the supernatant was collected, and 10% of the volume was used for biochemical investigation of the oxidative stress levels. The GSH (mg/mg), NO (μmol/mg), CAT (U/mg), and SOD (U/mg) levels were measured via ELISA kits according to the methods of Jollow et al. (25) and Buege and Aust (26). The optical density was assessed at 450 nm via a microplate reader.

### 2.9 Statistical analysis

The disparities were examined via one-way analysis of variance (ANOVA) in the SPSS Vision 21 statistical program. We employed the Duncan test to compare the mean values of the various substances. A probability of < 0.05 was deemed significant (*p* ≤ 0.05). We included the mean values alongside the standard deviation (mean±SD).

## 3 Result

**Figure 1:**
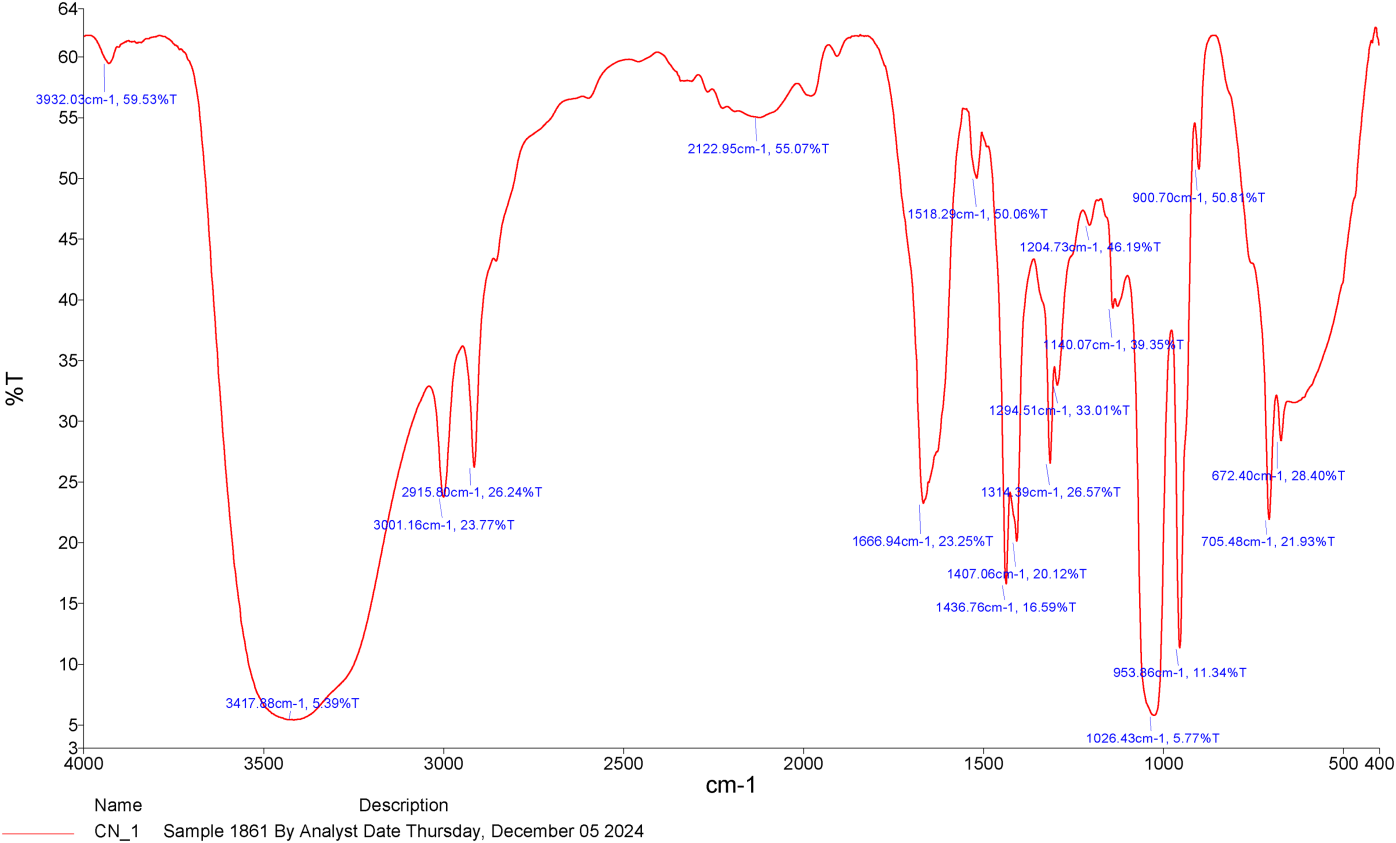
The functional characteristics of the active chemical compounds according to the FT-IR chromatograms of the *Cinnamomum verum* bark extract in methanol.

Chemical analysis was performed via Fourier transform infrared spectroscopy (FTIR), and the results revealed that the ethanolic CNBV included 18 chemical compounds, with major absorption bands between 3932.03 cm − 1 and 672.40 cm − 1 (Figure 1). The analysis revealed the following active phytochemical components: O-H stretching, N-H stretching, O-H stretching, N-H stretching, N=N=N stretching, C=O stretching, N-O stretching, C-H bending, O-H bending, O-H bending, C-O stretching, C-O stretching, C-O stretching, C-O stretching, and C=C bending, with an absorbance of 400–4,000/cm − 1 (Table 1).

**Table 1:** Fourier transform infrared spectroscopy of *Cinnamomum verum* bark extracts in the frequency range.

Absorption (cm^−1^)	Appearance	Transmittance (%)	Groups	Compound class
3932.03	Medium sharp	59.53	O-H Stretching	Alcohol
3417.88	Medium	5.33	N-H Stretching	Primary amine
3001.16	Weak, broad	23.77	O-H Stretching	Alcohol
2915.80	Strong, broad	26.24	N-H Stretching	Amine salt
2122.95	Strong	55.07	N=N=N Stretching	Azide
1666.94	Strong	23.25	C=O Stretching	Conjugated etone
1518.29	Strong	50.06	N-O Stretching	Nitro compound
1436.76	Medium	16.59	C-H Bending	Alkane
1407.06	Medium	20.12	O-H Bending	Carboxylic acid
1314.39	Medium	26.57	O-H Bending	Phenol
1294.51	Strong	33.01	C-O Stretching	Aromatic ester
1204.73	Strong	46.19	C-O Stretching	Alkyl aryl ether
1140.07	Strong	39.35	C-O Stretching	Tertiary alcohol
1026.43	Strong	5.77	C-O Stretching	Secondary alcohol
953.86	Strong	11.34	C=C Bending	Alkene
900.70	Strong	50.81	C-H Bending	1,2,4 risubstituted
705.84	Strong	21.93	C-H Bending	Benzene erivative
672.40	Strong	28.40	C-H Bending	Benzene

**Figure 2:**
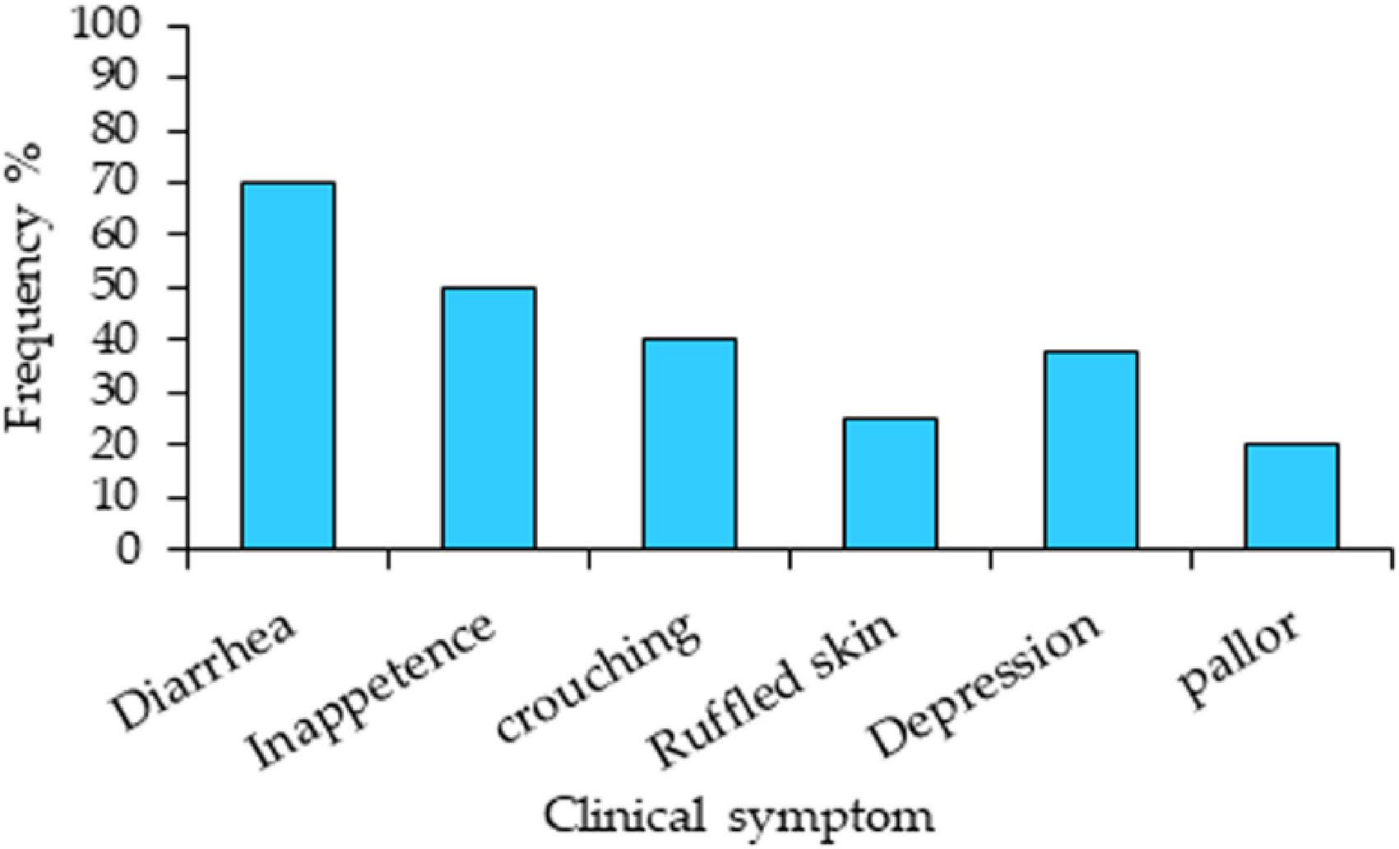
Clinical symptoms and their frequency in mice experimentally infected with *Eimeria papillata* without medication.

Figure 2 illustrates the frequency of clinical symptoms observed in the mice. The clinical indications varied in intensity among the experimental mice. The most prevalent sign was diarrhea. Infected mice clearly displayed coccidiosis symptoms, such as crouching, inappetence, sadness, and ruffled skin, whereas uninfected or amprolium-treated mice did not.

Table 2 shows the effects of CNB on the inhibition rate, incidence of bloody diarrhea, lesion score, and jejunal length for each therapy at 5 days postinjury. Pathological lesions in groups categorized from normal to severe. The scores of the infected group are shown in Table 2. It presents the length of the jejunum and the scores of the caeca, including jejunal atrophy, wall thickening, erosion, and black blood clots, which exhibited significant pathological abnormalities. Compared with those in the infected group, the jejunal morphology and length were considerably (*p* < 0.05) greater in the drug-treated group. When all the pathological parameters were aggregated, the lesion scores indicated that the herb-treated groups presented a significant (*p* < 0.05) reduction in unfavorable jejunal lesions induced by coccidiosis with increasing quantities of CNB extract (Table 2).

**Table 2:** Effects of the experimental groups on the incidence of diarrhea, lesion score, and jejunum length on the 5th day postinjury (5th DPI).

Groups	Bloody diarrhea	Lesion scores	Jejunum length%	Mortality %
Noninfected (−CVBE)	0^d^	0^d^	20.97^a^	0^d^
Noninfected +100 mg/mLCVBE	0^d^	0^d^	21.56^a^	0^d^
Infected (−CVBE)	1.45^b^	2.1^b^	18.54^b^	1^b^
Infected+100 mg/mLCVBE	0.3^c^	0.9^c^	22.56^a^	0^d^
Amproluim 50 mg/mL	0.2^c^	0.8^c^	22.11^a^	0^d^
Standard error	0.65	0.75	1.56	0.43
*p*-value	< 0.001	< 0.001	<0.005	<0.005

^a–c^Distinct lowercase letters inside the columns signify substantial variances.

Herb exhibited the fewest pathological characteristics, including the lowest lesion scores, with increasing dosage. Compared with the infected group, all the groups that were administered CNB had significantly lower lesion ratings (*p* < 0.05). No mortality occurred in any of the experimental groups, except for one mouse in the infected group. Consequently, the death rate was 4% across all the experimental groups. Neither the amprolium-treated nor the uninfected groups experienced diarrhea throughout the study. No bloody diarrhea was present in the feces during the initial two days post infection. All infected groups, except the amprolium-treated group, experienced bloody diarrhea between days 3 and 5 following infection with *E. papillate.*

We have clarified that the lesion score in Table 2 represents the total lesion severity, which was calculated by summing the scores of specific jejunal lesions (atrophy, wall thickening, mucosal erosion, and hemorrhage) graded according to a standardized 0–4 scale. The total lesion score presented in Table 2 represents the sum of all individual lesion grades for each bird, with higher values indicating greater lesion severity.

**Figure 3:**
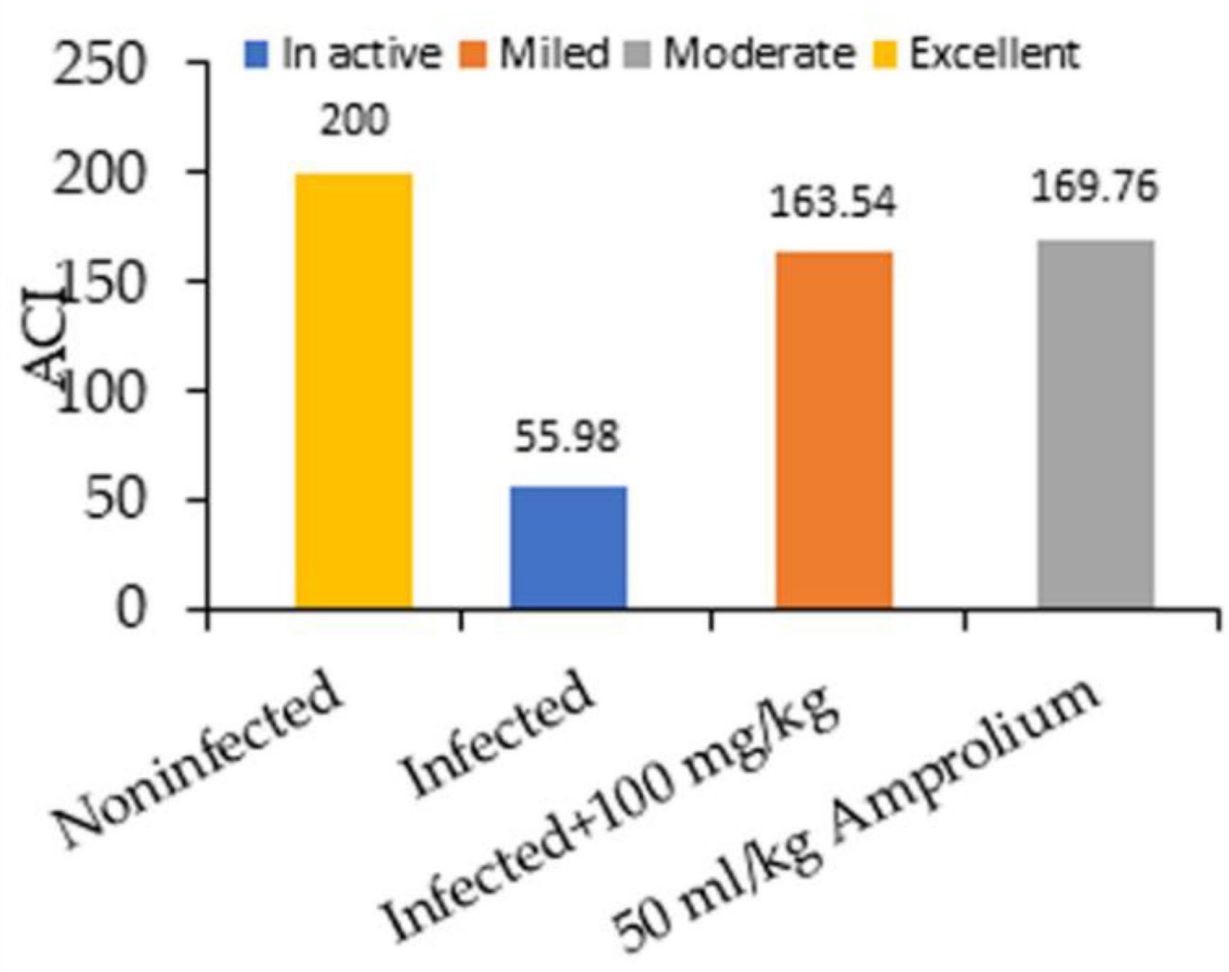
Impact of experimental groups and coccidiosis on the anticoccidial index (ACI) of each group five days post inoculation.

Figure 3 shows the impact of the experimental groups on the anticoccidian index (ACI) of each group five days post dosage. We administered 100 mg/kg CNB and 50 mg/kg amprolium to both the infected groups. ACI scores ranging from 120–140, 140–160, 160–180, or over 180 were categorized as mild, moderate, marked, or outstanding, respectively.

**Figure 4:**
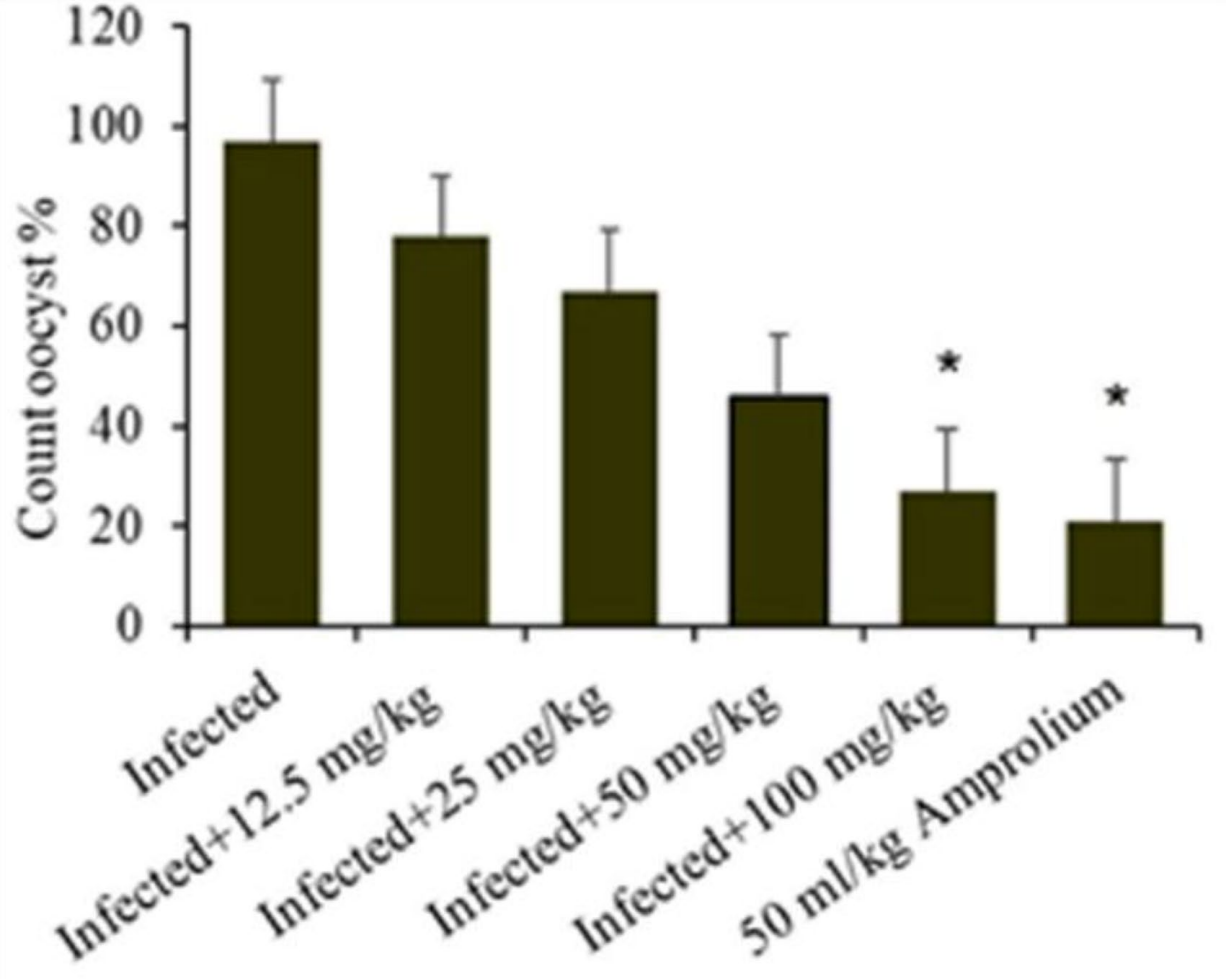
Shows the effect ratio of experimental *Cinnamomum verum* bark extracts on oocyst production on the fifth day following *Eimeria papillata* oocyst infection. Significance is relative to the infected cohort (*p* < 0.05).

**Figure 5:**
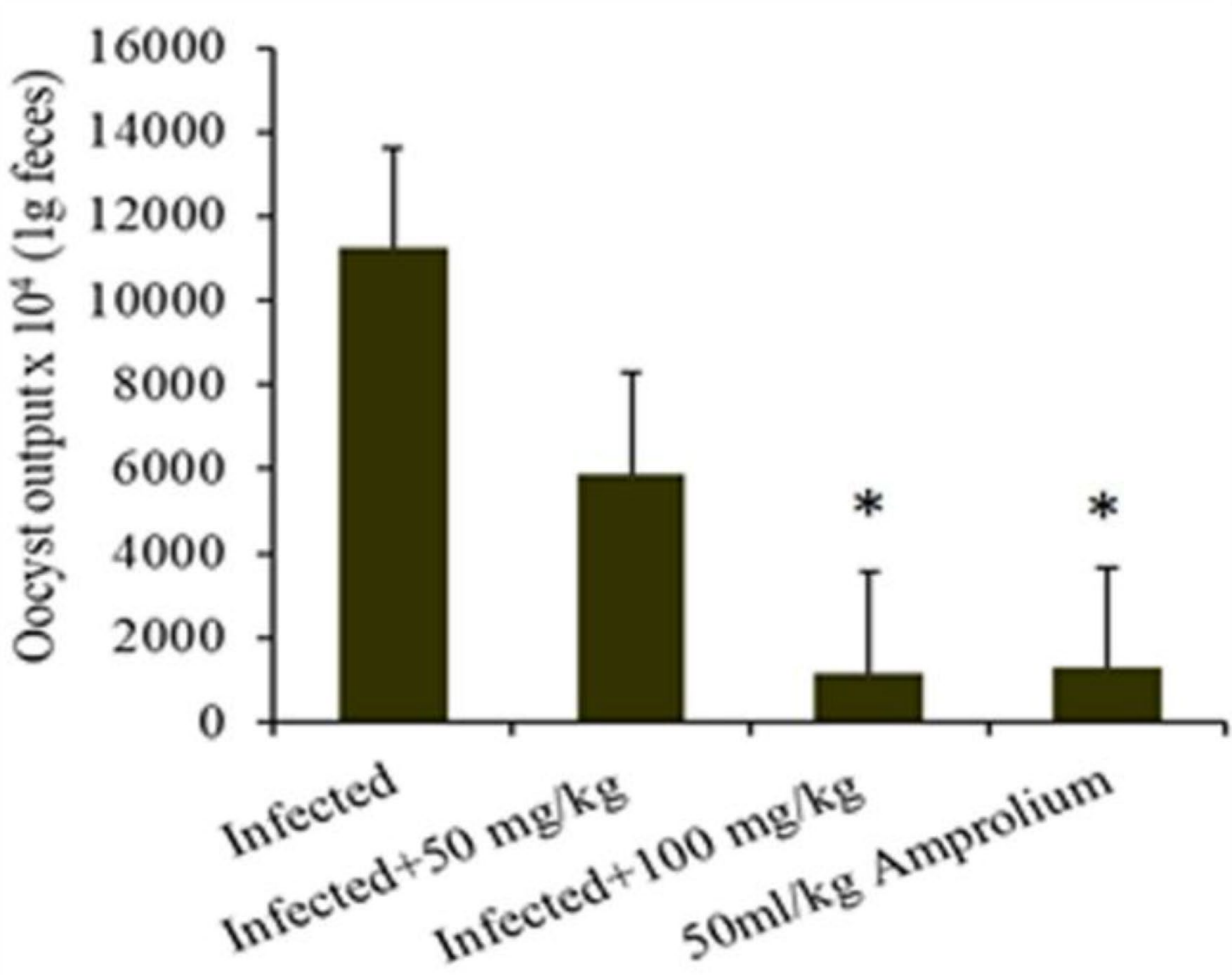
Illustrates the effect ratio of the experimental *Cinnamomum verum* bark extract sporulation oocyst count in 1 g of feces from infected, treatment (CVBE), and amprolium-treated mice. * The difference between the infected treated groups was significant at *p* ≤ 0.05.

In general, the ratio of oocyst excretion was significantly (*p* ≤ 5) affected by the experimental groups, which decreased with concentrations of 100 mg/kg of extract and 50 mg/kg amprolium. High oocyst excretion ratios were detected at 12.5, 25, and 50 g/L (Figure 4). Infection of the mice with *E. papillata* resulted in oocyst production peaking at approximately 88% per gram of feces in the infected cohort. In the groups administered *C. verum* bark extracts, oocyst generation decreased by 34, 49, 64, and 66% at dosages of 12.5, 25, 50, and 100 mg/kg extract, respectively, alongside 50 mL/kg reference medication (Figure 5), indicating a considerable reduction. The 100 mg/kg dosage had the greatest capacity to suppress fecal oocyst excretion. Therefore, it was exclusively used in subsequent studies.

*E. papillate* coccidian infection in mice led to oocyst excretion in fecal pellets, peaking at 228.75 × 10^3^ ± 54.66 oocysts/g of feces on day 5 post infection in the infected cohort. The oocyst ejection rate following treatment with 100 mg/kg *C. verum* bark extract was 32.17 × 10^3^ ± 5.25 oocysts/g feces, whereas it was 21.476 × 10^3^ ± 5.42 oocysts/g feces in the control group.

**Figure 6:**
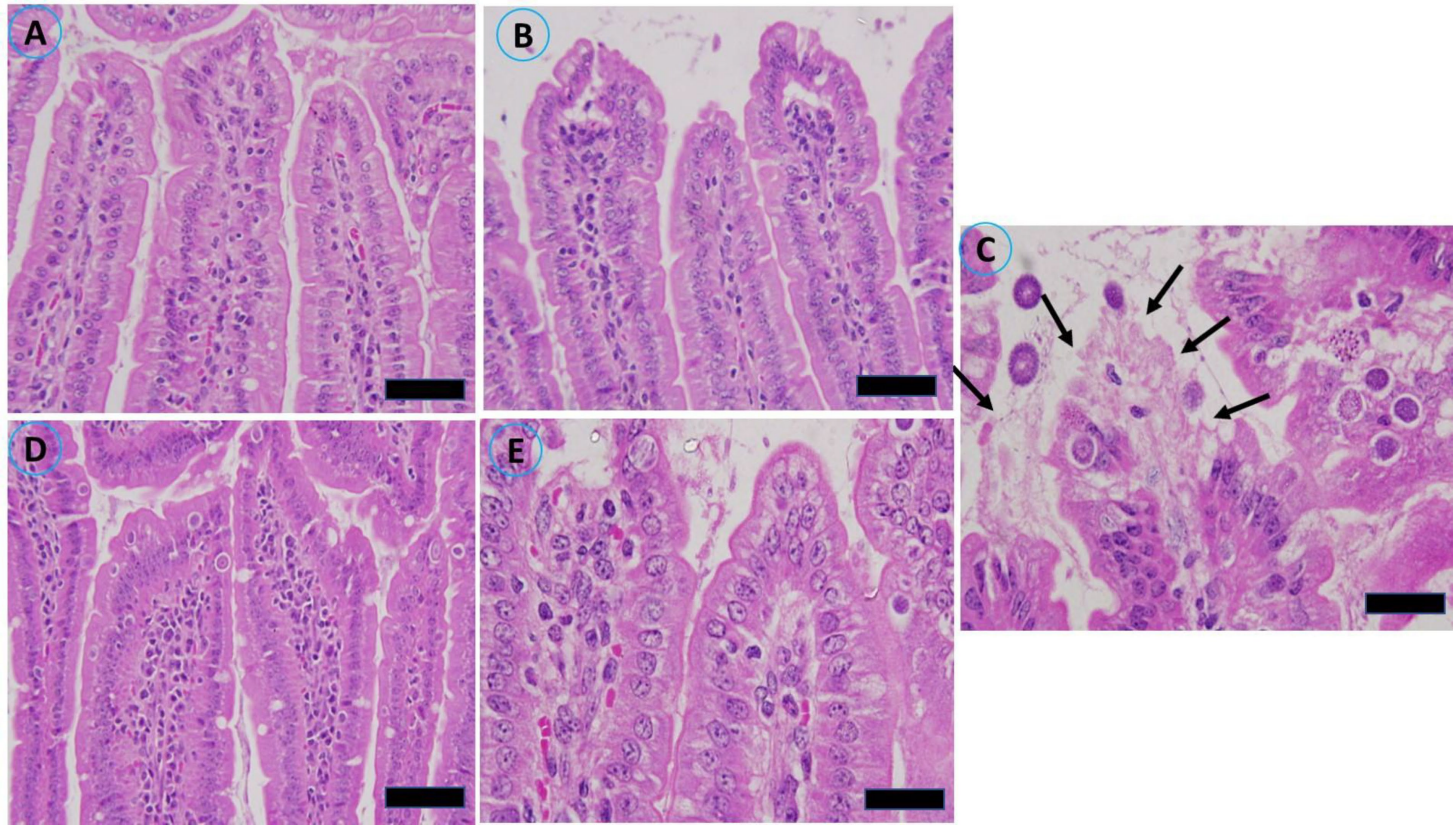
Histopathological alterations in the jejunal sections of mice resulting from infection with sporulated oocysts of *Eimeria papillata*, stained with Masson’s trichrome on day 5 post infection. **(A)** Noninfected (−CVBE); **(B)** Noninfected (+CVBE); **(C)** infected (−CVBE) denoted by arrows; **(D)** infected+100 mg/mL CVBE; **(E)** infected + amproluim 50 mg/mL. Scale Bar 20 μm.

Masson’s trichrome staining was performed on a segment of the murine jejunum to investigate the pathological alterations induced by infection of the jejunum. Histological examination via light microscopy revealed significant structural alterations in the gut wall. Prior to the administration of the extract, significant pathological abnormalities were observed in the jejunum of the infected group. The alterations included hemorrhaging in the intestinal villi, desquamation at the tips of the intestinal villi, and exfoliation of mucosal epithelial cells, alongside the emergence of parasite stages. Compared with the uninfected control mice, the infected group presented a marked reduction in villus height and crypt depth (Figure 6).

**Figure 7:**
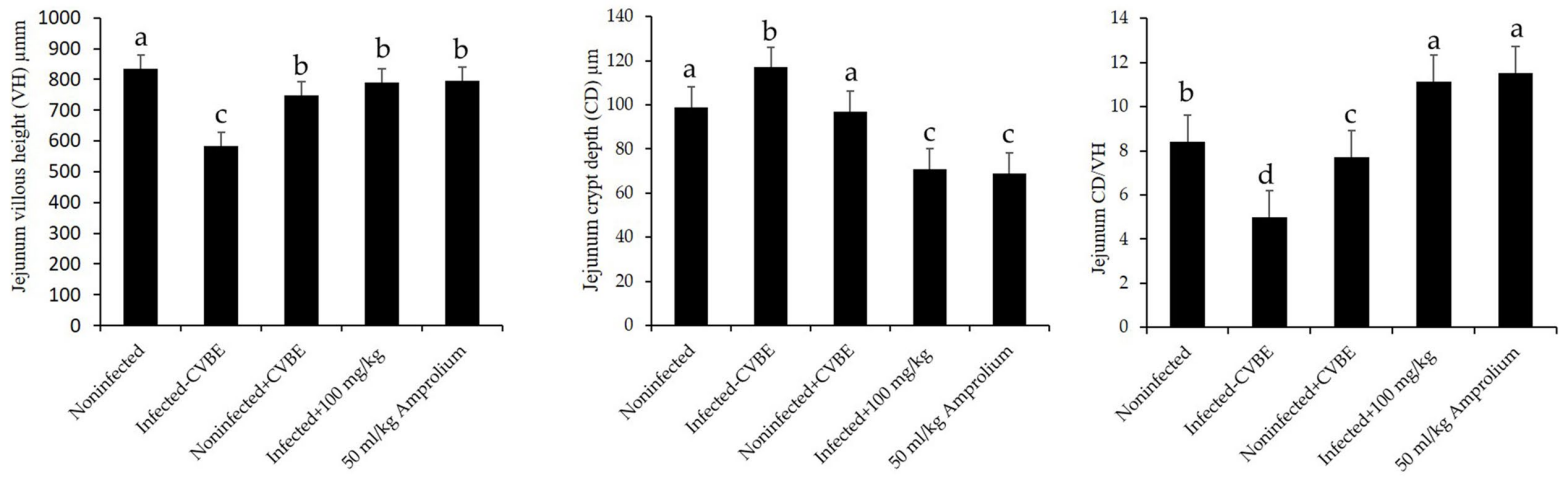
Impact of anticoccidial agents on jejunal histomorphometry in mice infected with *Eimeria papillata*. Noninfected group; infected group; noninfected group (+CVBE); infected with 100 mg/mL CVBE; infected with 50 mg/mL Amprolium. Data are presented as the mean values accompanied by their standard errors. Values denoted by distinct superscript letters are significantly different (*p* < 0.05).

Figure 7 shows that jejunal villous height (VH) was significantly lower in the infected group than in the noninfected group, crypt depth (CD) significantly affect. Anticoccidial treatments with 100 mg/mL of CVBE and 50 mg/mL Amprolium enhanced jejunal architecture in challenged mice; the villus height was greater in these groups than in the infected group, whereas the crypt depth was lower, and the VH-to-crypt depth ratio was greater in the CVBE and amprolium groups than in the other groups (Figure 7).

In Table 3, the results are presented as the average (SE), which represents the influence of *C. verum* bark extracts on the outcome of *E. papillata* infections. There was no fecal output of oocysts during the first-days post infection. On the fourth day the oocyst began to fall out, and in five-day post infection the highest oocyst output was 88.75 × 10^3^ ± 54.66 oocysts/g of feces in infected mice. When the mice were infected with *E. papillata*, the number of goblet cells in their jejunum decreased from 84.53 ± 4.56 to 59.84 ± 3.62 after treatment with *C. verum* bark extract (Table 3).

**Table 3:** Effect of CVBE on oocyst output on day five post infection with *Eimeria papillata* oocysts.

Treatment	Oocyst output/g feces	No. goblet cells/10 villi
Noninfected (−CVBE)	00 ± 00	84.53 ± 4.56
Noninfected (+CVBE)	00 ± 00	81.98 ± 3.87
Infected (−CVBE)	88.75 × 10^3^ ± 54.66	28.89 ± 2.32
Infected+100 mg/mL CVBE	32.17 × 10^3^ ± 5.25*	59.84 ± 3.62
Infected+Amproluim 50 g/mL	21.47 × 10^3^ ± 5.42*	62.69 ± 3.78

**Figure 8:**
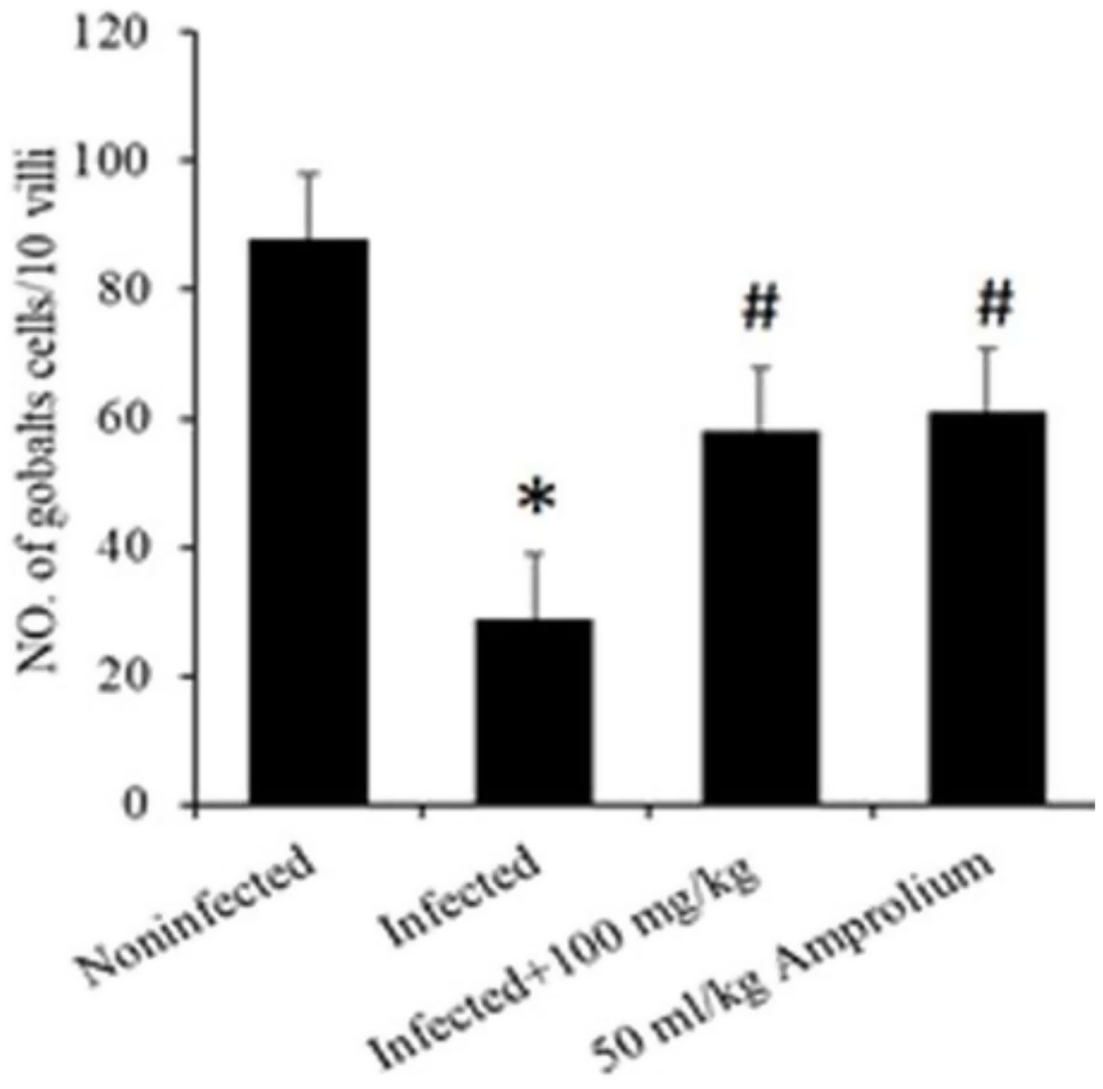
Impact of *Cinnamomum verum* bark extracts on the decrease in the goblet cell population in the jejunum of *Eimeria papillata*-infected mice. All values are shown as the means ± standard errors. Significant divergence from the control group (*p* ≤ 0.05) is indicated by *, #.

**Figure 9:**
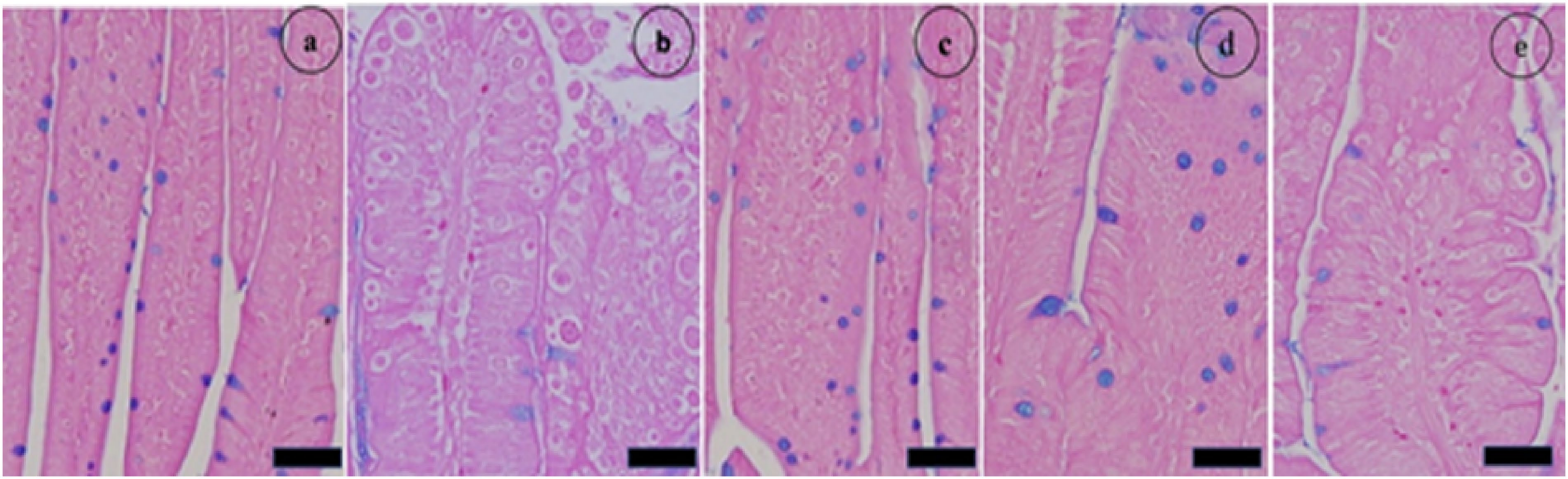
The impact of *Cinnamomum verum* bark extracts on the number of goblet cells in the jejunum of mice infected with *Eimeria papillata*. **(a)** denotes the noninfected group; **(b)** signifies the nontreated group exhibiting developmental stages; **(c)** indicates the noninfected group receiving *Cinnamomum verum* bark extracts only; **(d)** represents the treated group administered 100 mg/kg *Cinnamomum verum* bark extracts; and **(e)** refers to the infected group that received amprolium therapy. Alcian blue was used to stain the pieces. The scale bar represents 20 μm.

Microscopic investigation of Alcian blue-stained jejunal sections revealed that *E. papillata* infection significantly reduced the number of goblet cells in the jejunum (*p* < 0.05) compared with that in the noninfected group. Conversely, compared with those in the infected group, the number of goblet cells in the jejunum of the mice that were administered CVBE significantly increased (Figures 8, 9).

**Figure 10:**
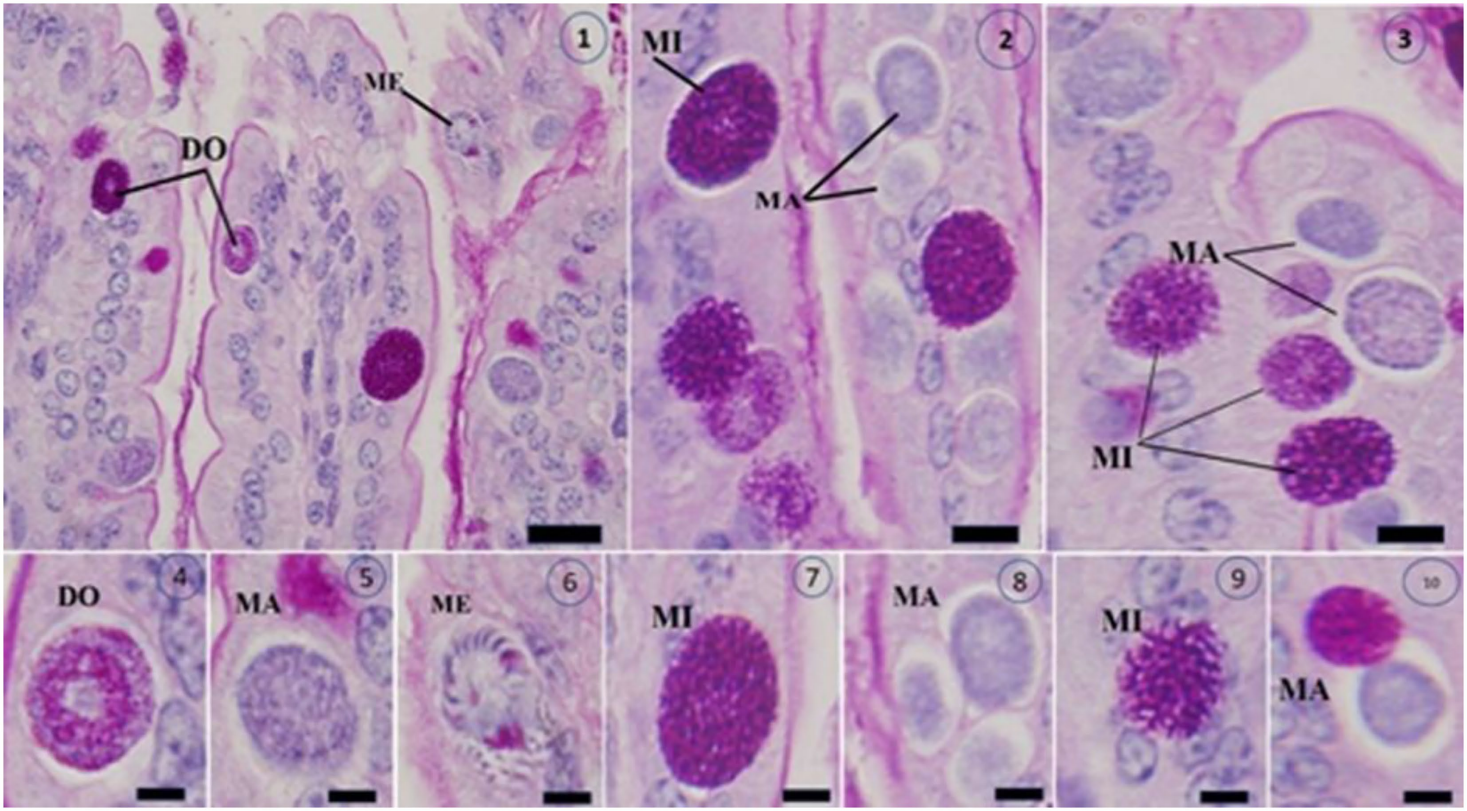
Shows Masson’s trichrome-stained slices of the jejunum infected with *Eimeria papillata* on day 5 after infection, revealing several developmental stages (1–10). The image displays a Meront (ME), macrogamont (MA), microgamont (MI), and developing oocyst (DO). Scale bar = 20 μm.

Figure 10 shows sections of jejunal villi stained with Masson’s trichrome. The administration of different dosages of *C. verum* bark extracts markedly reduced the total number of intracellular *E. papillate* stages, including the meronts, gamonts, and developing oocysts, especially at a dosage of 100 mg/kg *C. verum* bark extracts and reference drugs. This phenomenon was particularly evident in the mice that were administered extracts to address their disease. The epithelial cells in the jejunum of the mice infected with *E. papillate* presented different stages of parasite development. Compared with infection, treatment with *C. verum* bark extracts led to a substantial reduction of 69% in the number of parasite stages per ten villous–crypt units (*p* < 0.05).

Compared with that in the control group, *E. papilate* parasite infection led to a decrease in weight in the infected group, whereas the weight in the treated groups showed minimal negative or positive effects (Table 4).

**Table 4:** Mouse weight (g) at the beginning of the study (0–5 days).

Treatment	Weight of mice (g)		
	0 day	5 day	End - start (weight)
Noninfected	21.0325	20.295	0.7375 ± 0.37
Noninfected+ CVBE	21.43	21.83	0.3900 ± 0.32
Infected	20.5125	21.1375	−0.625 ± 0.31
Infected+100 mg/kg	20.76	20.6075	0.1525 ± 0.076
50 ml/kg Amprolium	21.435	21.265	0.17 ± 0.085

The infection disturbed the oxidative balance in the jejunum. A notable alteration occurred in the infected and treated mice. This was demonstrated by the evaluation of GSH levels, NO concentrations, and CAT activity.

**Figure 11:**
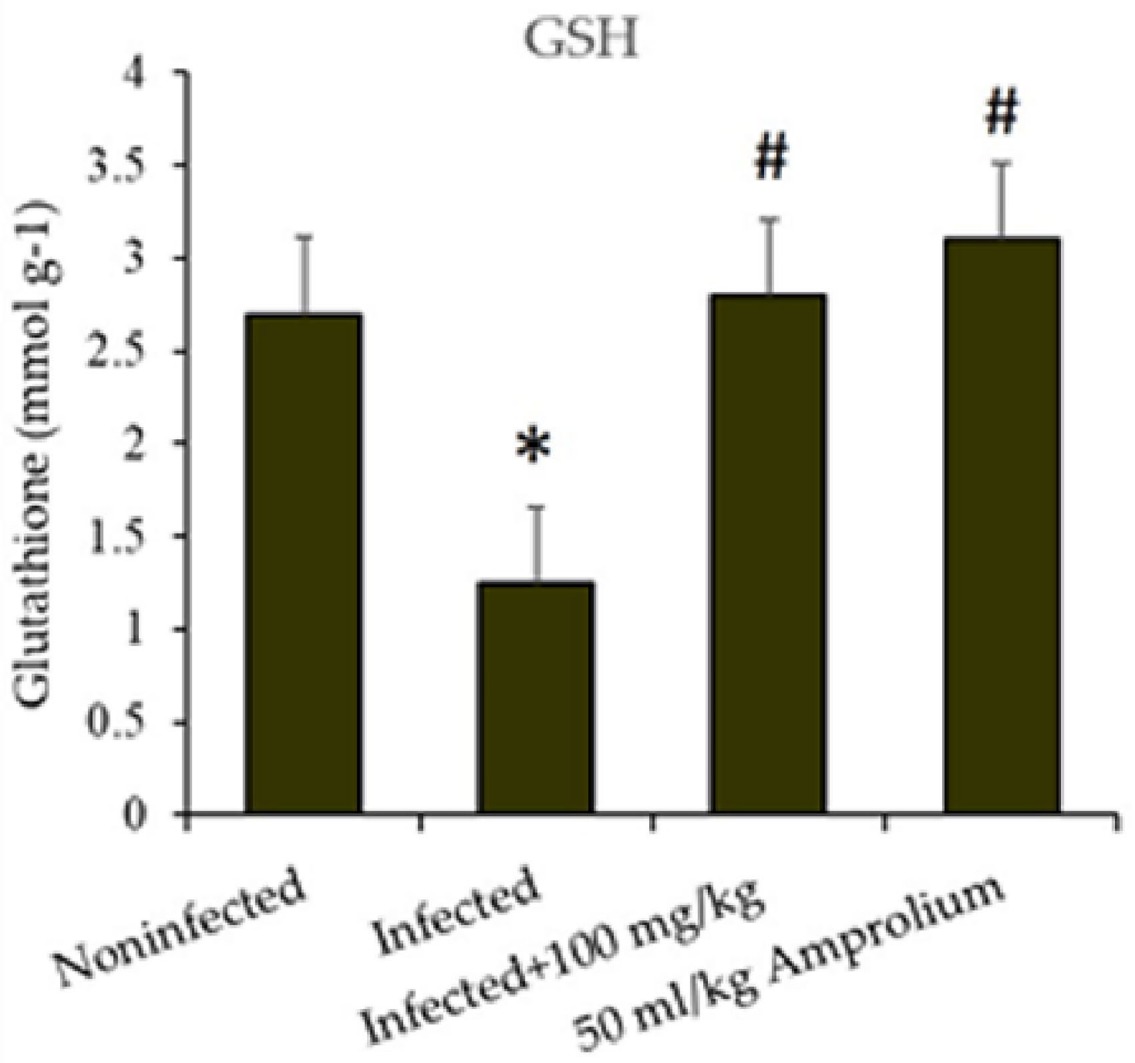
Impact of *Cinnamomum verum* bark extracts on jejunal glutathione levels in mice infected with *Eimeria papillata*. * and # indicate statistically significant differences between the control and infected groups, respectively, at *p* < 0.01.

*E. papillata* infection resulted in a substantial reduction (*p* < 0.01) in GSH levels in the jejunum of the mice. The reduction exceeded 40%; however, following the administration of *C. verum* bark extracts (100 mg/kg) to the sick mice, the GSH levels increased to nearly match those of the control groups. Compared with that of the reference medicine amprolium, the concentration of GSH was greater following treatment with the extracts (Figure 11).

**Figure 12:**
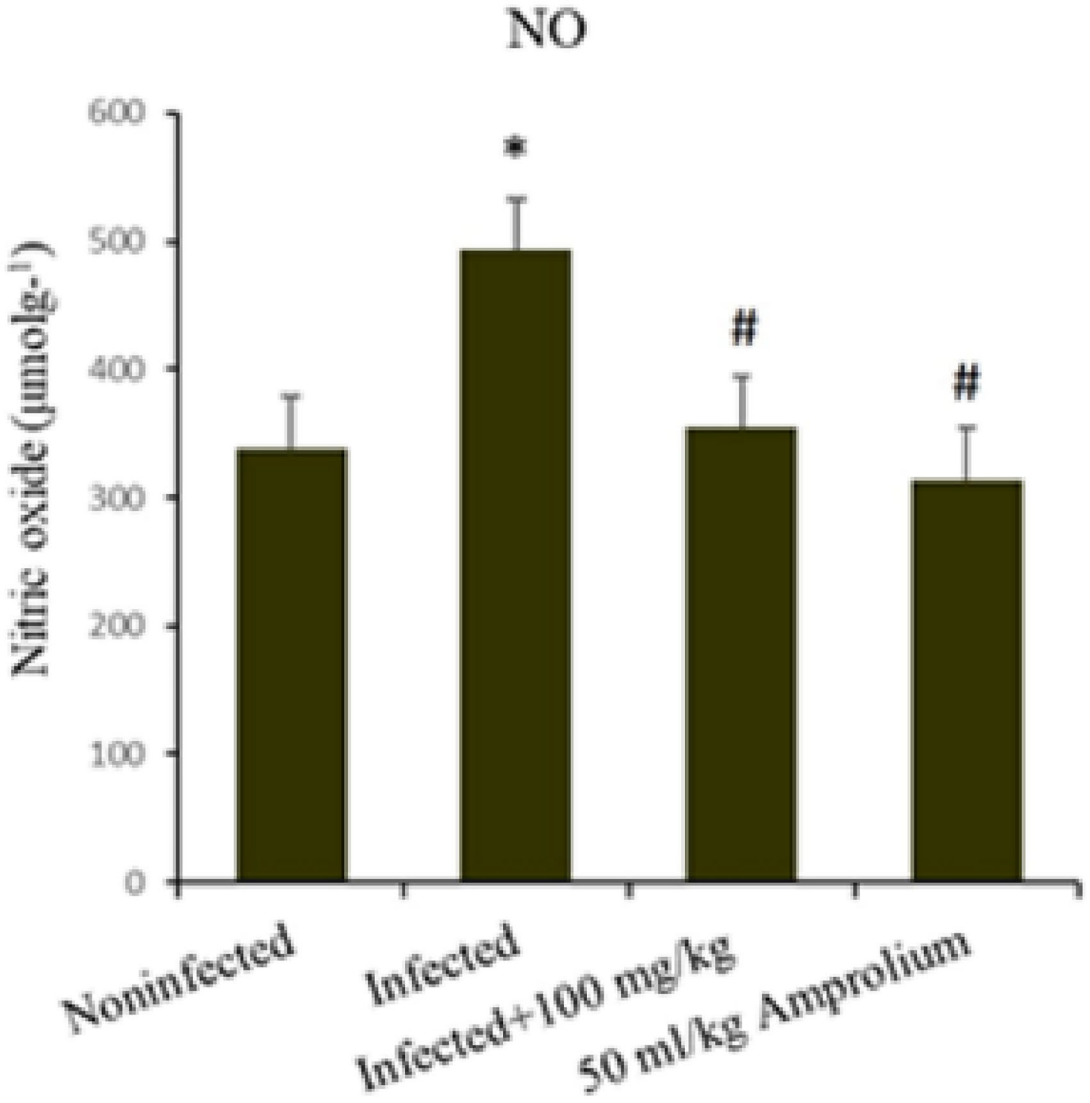
Impact of *Cinnamomum verum* bark extracts on jejunal nitric oxide levels in mice infected with *Eimeria papillata*. * and # indicate statistical significance in relation to the infected group at *p* < 0.01.

As shown in Figure 12, *E. papillata* infection resulted in a substantial increase (*p* < 0.01) in nitric oxide levels in the jejunum. This increase was mitigated following the treatment of infected mice with extracts of *C. verum* bark, resulting in nitric oxide levels approaching those of the noninfected control groups. Compared with the reference medicine amprolium, the concentration of nitric oxide was greater following treatment with the *C. verum* bark extracts (Figure 12).

**Figure 13:**
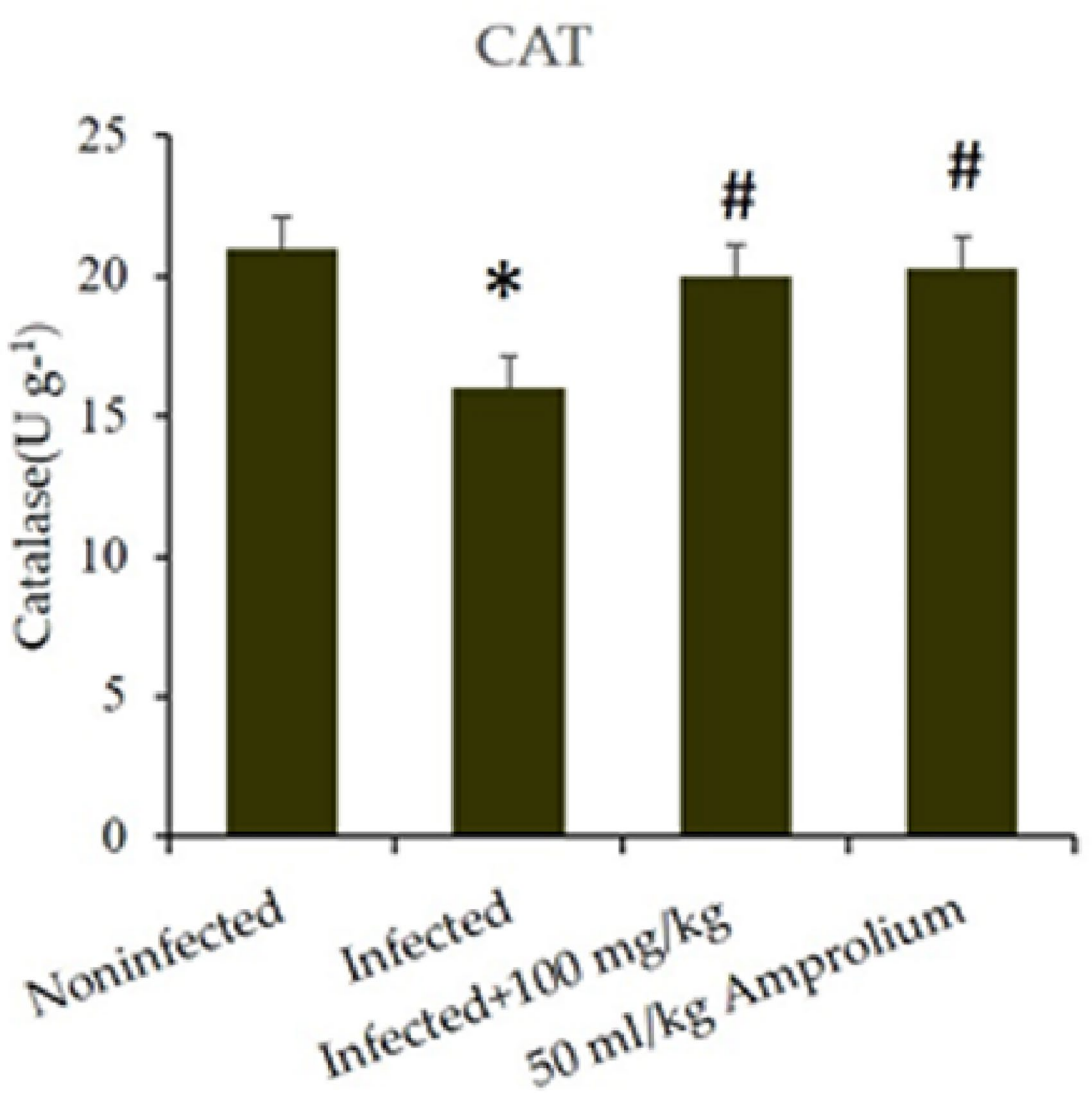
Impact of *Cinnamomum verum* bark extracts on jejunal catalase levels in mice infected with *Eimeria papillata*. * and # indicate statistical significance in relation to the infected group at *p* < 0.01.

In the jejunum of infected mice, catalase enzyme activity was considerably diminished (*p* < 0.01) following infection with *E. papillata*. *C. verum* bark extracts increased catalase activity to match that of the control (Figure 13).

## 4 Discussion

Amprolium functions as an anticoccidial ionophore that disrupts ion gradients across the membranes of Eimeria cells (27). The pursuit of synthetic anticoccidial alternatives is a vital research domain (22, 28, 29). We endeavored to replicate a synthetic anticoccidial by concentrating on identifying a conventional product exhibiting anticoccidial properties that are characterized by a distinctive, safe formulation devoid of tissue residues or drug resistance. Upon examining many plants, we concentrated on CNGE.

The many phytochemicals in specific herbs impart numerous bioactive characteristics to this shrub and spice (30). Initially, we concentrated on identifying a natural substance with anticoccidial qualities that may be further developed into a novel anticoccidial medication. Upon examining various herbs, we concentrated on the bark of *C. verum*. This spice contains a wealth of chemicals, such as cinnamaldehyde, cinnamate, cinnamic acid, alkaloids, flavonoids, various steroids, fatty acids, peptides, and several essential oils (31–33). It significantly lowers oocyst levels. Our data substantiate that the CNBE extract possesses considerable anticoccidial activity. The clinical manifestations of coccidiosis noted in the mice in this investigation included pallor, diarrhea, anorexia, depression, and ruffled fur, which aligns with other studies (34). In this trial, the incidence of bloody diarrhea in the infected treated groups was quantitatively lower than that in the infected groups. Consequently, diminished hemorrhagic diarrhea may safeguard diseased mice from secondary infections, inflammatory responses, and the consumption of hazardous agents (20). Cinnamon leaf extracts demonstrate a significant antidiarrheal effect in a castor oil-induced diarrhea model, confirming their efficacy in various diarrheal illnesses (29). Furthermore, cinnamon comprises active constituents, notably cinnamaldehyde, which enhances digestion and promotes the secretion of digestive enzymes (32, 35).

Consistent with other studies (21), the natural product significantly diminished the quantity of fecal oocysts in coccidia-infected mice. The herb-treated groups significantly diminished OPG production in the CNBE, which may be beneficial for managing extensive coccidiosis outbreaks on hen farms. The jejunum is one of the most essential digestive organs in mice. When *Eimeria* damages the epithelial cells of the jejunum, mice experience malabsorption and diarrhea, leading to reduced body weight gain (21). The extent of gross lesions and jejunal atrophy in the herb-treated groups varied from mild atrophy and dispersed petechiae to severe atrophy and extensive hemorrhaging in the infected group. Compared with those of the infected group, the data regarding jejunal lesions and oocyst reduction rates indicated that CNBE and amprolium effectively mitigated the problems associated with jejunal lesions (36).

These findings verify the presence of biologically active chemicals in CNGEs that impede or prolong the survival of *E. papillate in vivo*, leading to the suppression of lesions and oocyst excretion. Thus, cinnamon (37), which has direct antiparasitic effects on coccidian parasites, may significantly enhance the condition of afflicted mice due to its organ-protective attributes. Coccidiosis negatively impacted the affected group, which was mitigated by treatment with the natural product. The beneficial effects of cinnamon on anti-coccidiosis parameters may be attributed to the presence of substantial alkaloids, flavonoids, saponins, and tannins, which potentially exert synergistic effects on anti-parasitic, anti-inflammatory, antioxidant, and antidiarrheal activities in mice (30, 38). *Eimeria* infection in animals causes substantial weight loss (39), due to poor nutrition absorption and a reduced immunological response, resulting in intestinal damage (4). Our findings align with weight reduction in mice infected with *E. papillate* compared with their noninfected counterparts (6). This study corroborates the significant findings concerning the reduction in the number of oocysts and lesions in the jejunum; nonetheless, it has certain limitations. Although the 50 mg CNBE/kg dosage diminished the oocyst rate relative to that of the infected groups, it did not surpass that of the noninfected group. An overdose of coccidian oocysts or a subtherapeutic amount may be detrimental to the mice. The considerable potential effect of CNBE stems from the antioxidant and anti-inflammatory attributes of its constituent plant extract components (40, 41). Georgieva et al. (41), contend that coccidian infection disrupts the equilibrium between the body’s inherent antioxidant defenses and the generation of free radicals. These findings demonstrated that an *E. papillata* infection is associated with oxidative damage to the jejunum in mice, resulting in a decrease in antioxidant enzymes and GSH levels and CAT activity, while increasing NO concentrations. These oxidative parameters are crucial for safeguarding an animal’s body from free radical damage during Eimeria infection. Antioxidant enzymes employ the active sites of plant extracts to counteract the accumulation of reactive oxygen species that cause harmful changes in the jejunal epithelium (41). CNBE markedly inhibits the infection-induced depletion of these indicators and enhances their activity, which is generally diminished during oxidative damage caused by infection. This study utilized herbal extracts as anticoccidial agents to address *E. papillate* infection. This study revealed that CNBE therapy impedes the growth of this parasite, consequently resulting in an increase in the number of goblet cells due to damage. Murshed earlier, *Vitis vinifera* extract treatment exhibited significant efficacy and effectiveness against Eimeria (6). GSH functions as the initial line of defense against the harmful effects of reactive oxygen species (ROS), such as the toxicity associated with lipid peroxidation (42). This study indicates that the reduction in GSH observed in the jejunum of infected mice may signify increased consumption due to oxidative stress, suggesting that the intestinal lining is compromised by the Eimeria parasite. Nitric oxide synthases ultimately convert most of the nitric oxide produced in tissues into nitrate in bodily fluids (43). The increase in intestinal nitric oxide production is often linked to adverse consequences and is related to jejunal megaly (44). Catalase, an enzyme prevalent in living organisms, is essential for reducing oxidative stress by facilitating the breakdown of hydrogen peroxide (H_2_O_2_) into water and oxygen. This mechanism inhibits the buildup of reactive oxygen species (ROS) and subsequent oxidative damage to cells and tissues (45). This study evaluated catalase during E. papillate infection, as this enzyme directly controls certain free radicals, thereby diminishing them due to the reduced activity of the antioxidant defense system (46). CNBE demonstrates considerable anticoccidial efficiency. This is supported by studies demonstrating a reduction in oocyst excretion and sporulation, a decline in the stages of parasite proliferation in the jejuna, and a normalization of the goblet cell count.

## 5 Conclusion

Conclusions of *in vivo* studies have shown that these extracts markedly impede oocyst sporulation. Thorough pharmacological studies are essential for determining the new pharmacodynamic effects of bioactive components. Moreover, the essential mechanisms underlying these bioactivities require comprehensive examination. Clarifying the histological and molecular mechanisms behind sporulation suppression by CVBE and its protective effects against *E. papillata*-induced intestinal infection *in vivo* is crucial.

## Data Availability

The original contributions presented in the study are included in the article/supplementary material, further inquiries can be directed to the corresponding author/s.
